# Supplementary material for: Transferrin-Decorated Niosomes with Integrated InP/ZnS Quantum Dots and Magnetic Iron Oxide Nanoparticles: Dual Targeting and Imaging of Glioma
Source: Int J Mol Sci. 2021 Apr 27;22(9):4556. doi: 10.3390/ijms22094556 (PMC8123697; doi:10.3390/ijms22094556)
Supplement: Supplementary file 1 [file ijms-22-04556-s001.zip › ijms-1176514-SI.pdf]

Electronic Supplementary Information

Transferrin-Decorated Niosomes with Integrated InP/ZnS Quantum Dots and Magnetic Iron Oxide Nanoparticles: Dual Targeting and Imaging of Glioma

Didem Ag Seleci [1,2,†], Viktor Maurer [1,2,†], Firat Baris Barlas [3], Julian Cedric Porsiel [1], Bilal Temel [1], Elcin Ceylan [3], Suna Timur [3], Frank Stahl [4], Thomas Scheper [4] and Georg Garnweitner [1,2,*]

[1] Institute for Particle Technology (iPAT),Technische Universität Braunschweig, 38104 Braunschweig, Germany; d.ag-seleci@tu-braunschweig.de (D.A.S.); v.maurer@tu-braunschweig.de (V.M.); c.porsiel@tu-braunschweig.de (J.C.P.); b.temel@tu-braunschweig.de (B.T.)
[2] Center of Pharmaceutical Engineering (PVZ), Technische Universität Braunschweig, 38106 Braunschweig, Germany
[3] Faculty of Science Biochemistry Department, Ege University, 35100 Izmir, Turkey; f.b.barlas@gmail.com (F.B.B.); elcinceylan5@gmail.com (E.C.); suna.timur@ege.edu.tr (S.T.)
[4] Institute for Technical Chemistry | Leibniz University Hannover, 30167 Hannover, Germany; stahl@iftc.uni-hannover.de (F.S.); scheper@iftc.uni-hannover.de (T.S.)
* Correspondence: g.garnweitner@tu-braunschweig.de
† Contributed equally to the work.

Synthesis and characterization of InP/ZnS QDs

Briefly, to prepare the InP core particles, 0.19 g $InCl_3$ and 0.12 g $ZnCl_2$ was added to 30 mL OLA under stirring and the mixture evacuated at room temperature for 1 h and subsequently at 120 °C for 30 minutes. Under nitrogen atmosphere, the temperature was then increased to 225 °C. After the precursors were completely dissolved, 0.25 mL of TDMAP was injected into the solution to initiate particle formation. After 10 minutes the temperature was decreased to 200 °C and 1.79 g of DDT was slowly added to the solution to deploy a first layer of the ZnS shell. The reaction was continued for 1 h and then allowed to cool down. 10 mL of hexane was added and the solution was centrifuged to remove unreacted solid precursor. The supernatant was mixed with 200 mL of acetone and centrifuged again to precipitate the QDs. The product was dried with nitrogen gas and 0.24 g of zinc stearate as well as 20 mL of OLA added to dissolve the QDs. The solution was again evacuated at room temperature for 20 minutes, and heated to 185 °C for 2 h under nitrogen atmosphere. To yield the final product, 15 mL of hexane was added to the solution after cooling to room temperature, and the mixture centrifuged to remove unreacted zinc stearate. For the final purification, 200 mL of acetone was added to the supernatant, the product separated by centrifugation, dried with nitrogen gas and

redispersed in 30 mL of hexane. This purification step was repeated two more times, whereby the final product was redispersed and stored in 4 mL of chloroform.

Supplementary Figures

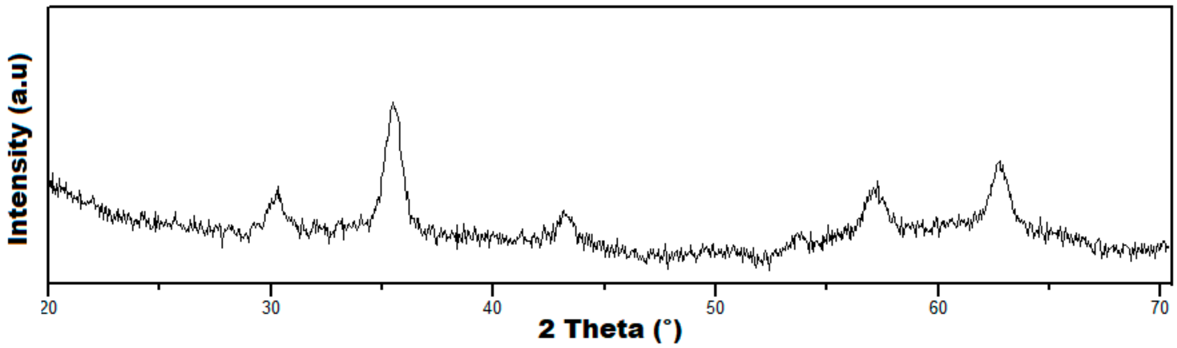

Figure S1. XRD pattern of the CA-MION nanoparticle powder.

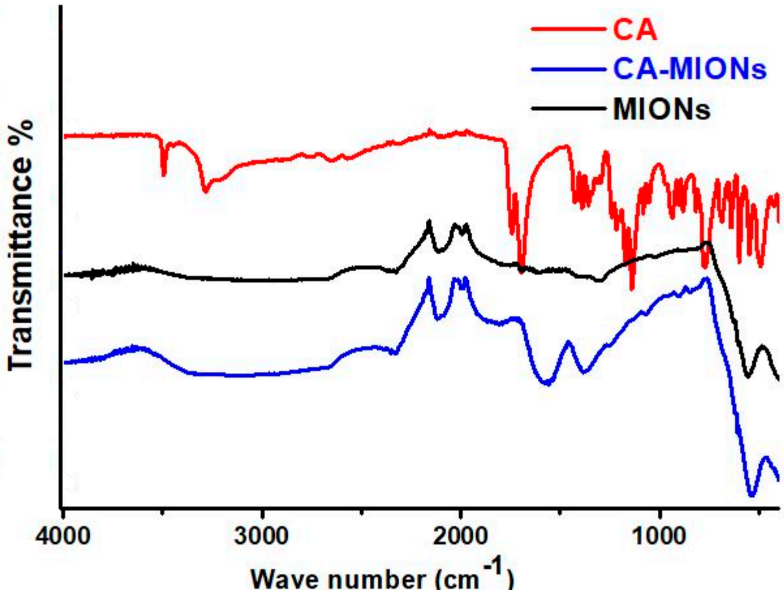

Figure S2. FTIR spectra of neat CA, MIONs and CA-MIONs.

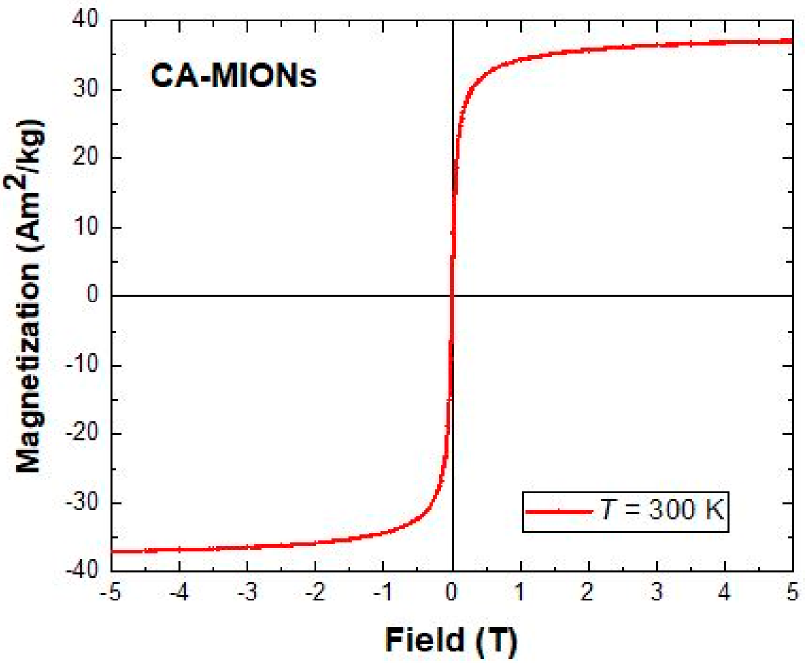

Figure S3. Magnetization curve of CA-MIONs.

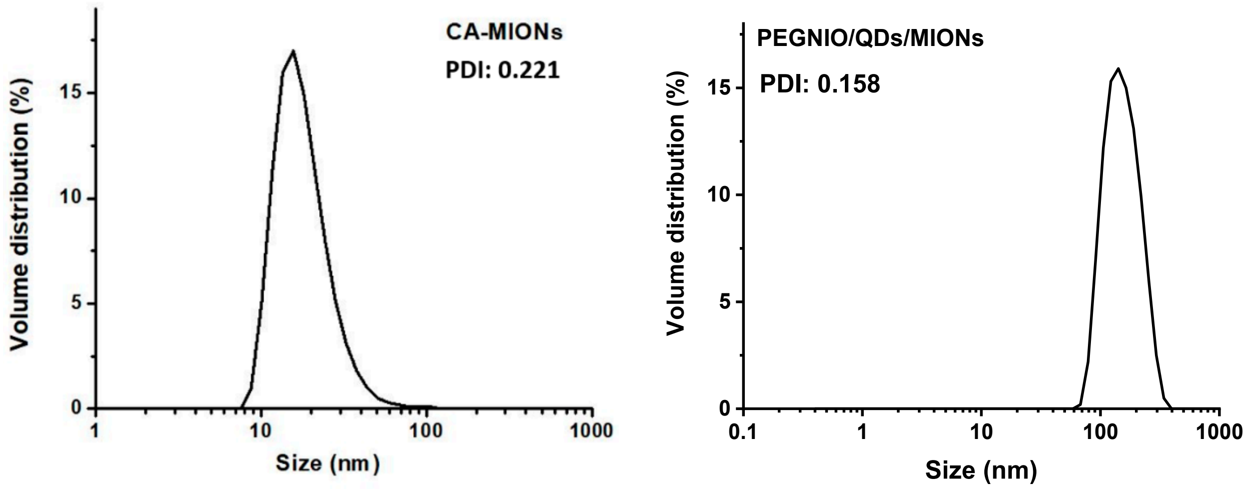

Figure S4. Dynamic light scattering measurements of CA-MIONs and PEGNIO/QDs/MIONs

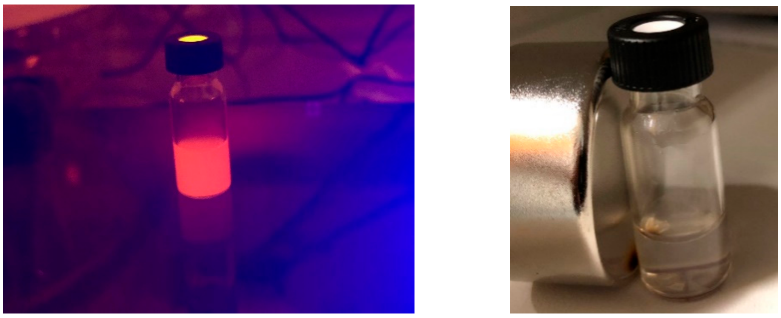

Figure S5. Image of PEGNIO/QDs/MIONs under UV light and migration of PEGNIO/QDs/MIONs after external magnetic field treatment.

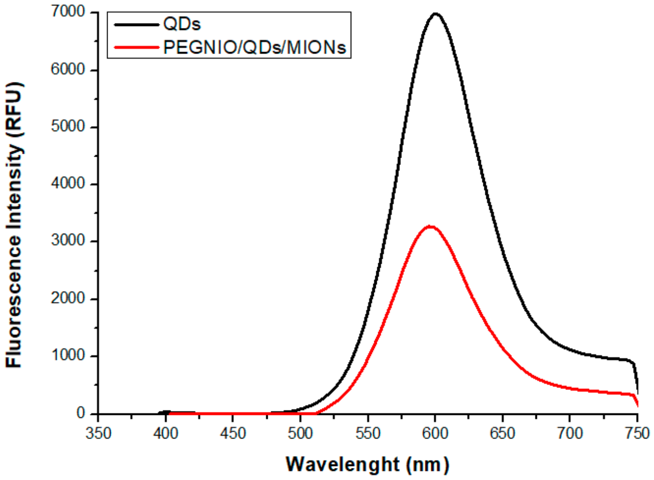

Figure S6. Fluorescence spectra of QDs and PEGNIO/QDs/MIONs.

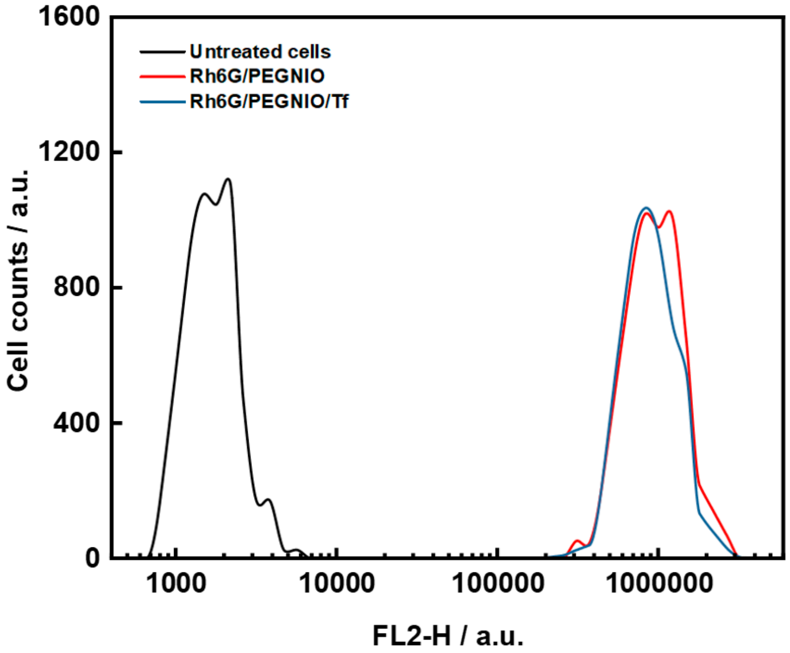

Figure S7. Flow-cytometric measurement of rhodamine 6G-labeled PEGNIO and PEGNIO/Tf uptake by Tf-receptor-negative A549 cells.
